# Expression and Significance of MicroRNA-183 in Hepatocellular Carcinoma

**DOI:** 10.1155/2013/381874

**Published:** 2013-10-10

**Authors:** Zenghui Liang, Yingtang Gao, Wenxia Shi, Daokuan Zhai, Shilei Li, Li Jing, Hua Guo, Tong Liu, Yajie Wang, Zhi Du

**Affiliations:** ^1^Third Central Clinical College of Tianjin Medical University, Tianjin 300170, China; ^2^Key Laboratory of Artificial Cell, Institute for Hepatobiliary Disease, Tianjin Third Central Hospital, Tianjin 300170, China; ^3^Department of Hepatobiliary Surgery, Tianjin Third Central Hospital, Tianjin 300170, China

## Abstract

*Objective*. In our previous study, we found that some miRNAs were deregulated in hepatocellular carcinoma (HCC), including miR-183. However, the expression of miR-183 in the progression of benign liver diseases to HCC and its correlation with clinicopathologic factors remain undefined. *Methods*. MiR-183 expression was measured in normal controls (NC) (*n* = 21), chronic viral hepatitis B or C (CH) tissues (*n* = 10), liver cirrhosis (LC) tissues (*n* = 18), HCC tissues (*n* = 92), and adjacent nontumor tissues (NT) (*n* = 92) by quantitative real-time reverse-transcription polymerase chain reaction (qRT-PCR). *Results*. The expression levels of miR-183 were significantly higher in HCC than in NT, LC, CH, and NL (*P* = 0.001, *P* < 0.001, *P* = 0.011, *P* < 0.001, resp.). The upregulated miR-183 in HCC was correlated with TNM stage (*P* = 0.042) and cirrhosis (*P* = 0.025). The Kaplan-Meier survival analysis showed that miR-183 expression was not associated with the survival of HCC patients. However, miR-183 yielded an area under the curve (AUC) of 0.808 with 59.8% sensitivity and 91.8% specificity in discriminating HCC from benign liver diseases (CH and LC) or NC. *Conclusions*. The upregulated miR-183 may associate with onset and progression of HCC, but not with the patient survival. A further research is needed to determine the potential of miR-183 as biomarker for HCC.

## 1. Introduction

Hepatocellular carcinoma (HCC) represents the major histological subtype of primary liver cancers [1], and it is one of the most common causes of cancer-related death in the world [2]. Many studies have shown that microRNAs (miRNAs) are deregulated in HCC. miRNAs are small noncoding RNA molecules that are involved in the regulation of gene expression and protein translation. Growing evidence indicates that their deregulation plays an important role in the pathogenesis and development of HCC [3].

It has been manifested that the deregulation of miR-183 is associated with clinicopathological factors (including metastasis, invasion clinical stage, and tumor size), and considered as the potential biomarker for diagnosis and prognosis of cancer patients [4–8]. Both Li et al. [9] and Liu et al. [10] reported that the expression of miR-183 was significantly upregulated in HCC tissues compared with the matching nontumor liver tissues. Goeppert et al. [11] reported that expression of miR-183 was significantly higher in LC, premalignant lesions, and HCC versus NC and that miR-183 reduced tumor suppressor gene in human hepatocarcinogenesis. In addition, Liu et al. [10] reported that serum miR-183 was derived primarily from tumor, but the sensitivity and specificity of serum miR-183 were insufficient to be biomarkers. Therefore, the expression of miR-183 and its correlation with clinicopathological factors still need further research.

Our previous study analyzed the differential expression of 88 miRNAs in 11 pairs of HCC and matched NT tissues by RT2 miRNA PCR array [12]. The results showed that miR-183 was the most significant upregulated miRNA in HCC patient (7.127-fold). Based on our previous study, we aimed to detect the expression of miR-183 in NC, CH, LC, NT, and HCC and investigate the correlations between miR-183 expression and clinicopathological factors, including patient survival.

## 2. Methods

### 2.1. Patients

In this study, HCC tissues and adjacent nontumor tissues were obtained from 92 patients with HCC who received hepatectomy, 18 tissue samples from patients with liver cirrhosis, 10 tissue samples from patients with chronic hepatitis B virus or C virus (HBV or HCV), and 21 tissue samples from hepatic hemangioma patients or hepatic abscess (normal controls). All samples were obtained from patients who had undergone surgery at Tianjin Third Central Hospital (Tianjin, China) from December 2003 to December 2009. All samples were examined by a pathologist and graded histologically. Samples were collected and stored at −80°C until analysis. 

All patients provided informed consent before enrollment in the study, and the study was approved by the Ethics Committee of Tianjin Third Central Hospital.

### 2.2. Follow-Up

The follow-up was completed on July 1, 2012. The period of follow-up was defined from the date of surgery to the date of patients death or the last follow-up point. All patients were monitored after surgery. The follow-up program included serum alpha-fetoprotein (AFP), abdominal ultrasound, enhanced computed tomography (CT), and magnetic resonance imaging (MRI). 

### 2.3. RNA Extraction

Total RNA was extracted from liver tissue using TRIzol Reagent (Invitrogen, Carlsbad, CA, USA) according to the manufacturer's instruction.

### 2.4. Reverse Transcription (RT)

Total RNA samples were reverse-transcribed to cDNA using TaqMan miRNA reverse transcription kit (Applied Biosystems, Foster, CA, USA). The 15 *μ*L RT reaction contained 0.15 *μ*L dNTP mix (100 mM total), 1 *μ*L Multiscribe RT enzyme (50 U/*μ*L), 1.5 *μ*L 10x RT buffer, 0.19 *μ*L RNase inhibitor (20 U/*μ*L), 4.16 *μ*L nuclease-free water, 3 *μ*L primer, and 5 *μ*L RNA. The reaction was carried out at 16°C for 30 min, 42°C for 30 min, and 85°C for 5 min on the ABI ViiA 7 Real-time PCR system (Applied Biosystems, USA).

### 2.5. Quantitative Real-Time Reverse Transcription Polymerase Chain Reaction (qRT-PCR) Analysis

The qRT-PCR was performed by TaqMan Universal PCR master mix (Applied Biosystems, Foster, CA, USA) on the ABI ViiA 7 real-time PCR system (Applied Biosystems, USA). Each amplification reaction was performed in volume of 20 *μ*L containing 10 *μ*L TaqMan Universal PCR master mix, 1 *μ*L TaqMan Assay (20x), 1.5 *μ*L cDNA template, and 7.5 *μ*L RNase-free water. Reaction was performed at 95°C for 10 min, 40 cycles of 95°C for 15 s, and 60°C for 60 s. Each sample was run repeatedly, and the mean of two results was used to make statistical calculation. The expression of miR-183 was calculated using 2^−ΔΔCT^ method.

### 2.6. Statistical Analysis

SPSS 17.0 software (SPSS. Chicago, IL, USA) was used for the statistical analysis. All data were presented as median (range, 25th and 75th percentiles). The Mann-Whitney *U*-test was used to compare the differences among the groups. The diagnostic value for differentiating between HCC patients and the controls was evaluated by receiver-operator characteristic (ROC). Overall and disease-free survival rates were calculated according to the Kaplan-Meier method and analyzed by the log-rank test. Univariate and multivariate analyses of the prognostic factors were performed with the Cox proportional hazard analyses. *P* < 0.05 was considered statistically significant.

## 3. Results

### 3.1. MiR-183 Expression in Normal Control and Diseased Liver Tissue

**Figure 1:**
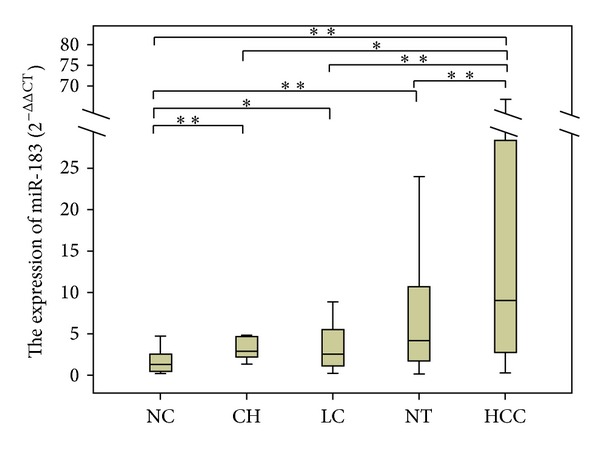
The expression levels of miR-183 in patients with liver diseases and normal control. *P < 0.05, **P < 0.001. The expression of miR-183 in HCC group was significantly higher than that in NC, CH, LC, and NT (*P* < 0.001, *P* = 0.011, *P* < 0.001, *P* = 0.001), and NC group was significantly lower than CH, LC, and NT (*P* = 0.008, *P* = 0.035, *P* < 0.001). There was no significant difference in CH, LC, and NT (*P* = 0.632, *P* = 0.345, *P* = 0.082).

The expression levels of miR-183 were 1.254 (0.415~2.592) in NC group, 2.825 (1.964~4.670) in CH group, 2.249 (0.868~4.821) in LC group, 4.121 (1.609~10.890) in NT group, and 9.015 (2.687~28.786) in HCC group. MiR-183 expression in HCC was significantly higher than that in NC, CH, LC, and NT. (*P* < 0.001, *P* = 0.011, *P* < 0.001, *P* = 0.001, resp.). MiR-183 expression levels in CH, LC, and NT were significantly higher than those in NC (*P* = 0.008, *P* = 0.035, *P* < 0.001, resp.), no significant differences were found among CH, LC, and NT (*P* = 0.632, *P* = 0.345, *P* = 0.082, resp.) (Figure 1).

### 3.2. The Differentiating Power of MiR-183 Expression in Patients with HCC and the Controls

**Figure 2:**
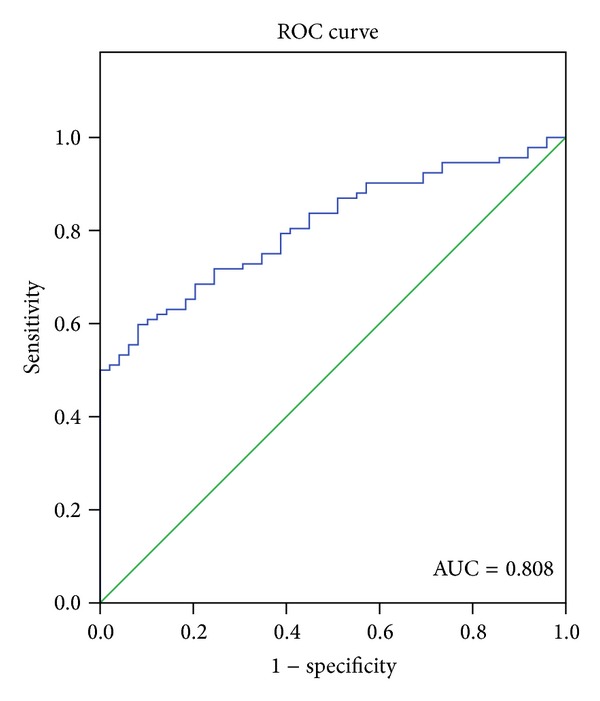
Receiver operating characteristics (ROC) curve of miR-183 to differentiate HCC patients from benign liver diseases or normal controls. The area under the curve (AUC) was 0.808 with 95% confidence interval (CI): 0.739–0.877. Optimal cutoff value was 5.81 (2^−ΔΔCT^) for miR-183, where the sensitivity and specificity were 59.8% and 91.8%, respectively.

The ROC curve analysis indicated that miR-183 was useful in differentiating HCC from benign liver diseases or normal controls. The area under the curve (AUC) of ROC was 0.808 with 95% confidence interval (CI): 0.739–0.877. At cutoff value of 5.81 (2^−ΔΔCT^), the sensitivity and specificity were 59.8% and 91.8%, and the positive predictive value and negative predictive value were 87.9% and 69.6%, respectively (Figure 2).

### 3.3. MiR-183 Correlates with Clinicopathological Factors of HCC 

The correlation between miR-183 expression and clinicopathological factors of the HCC patients was summarized in Table 1. The results revealed that the expression of miR-183 was significantly higher in HCC with cirrhosis (*P* = 0.025) and that TNM stage (III-IV) was higher than TNM stage (I-II) (*P* = 0.042). But there was no correlation between miR-183 expression and other clinicopathological factors, such as age, gender, HBV infection, HCV infection, *α*-fetoprotein levels, tumor size, tumor number, vein invasion, and histological grade (*P* > 0.05) (Table 1).

### 3.4. The Correlation between MiR-183 Expression and the Prognosis of HCC Patients

**Figure 3:**
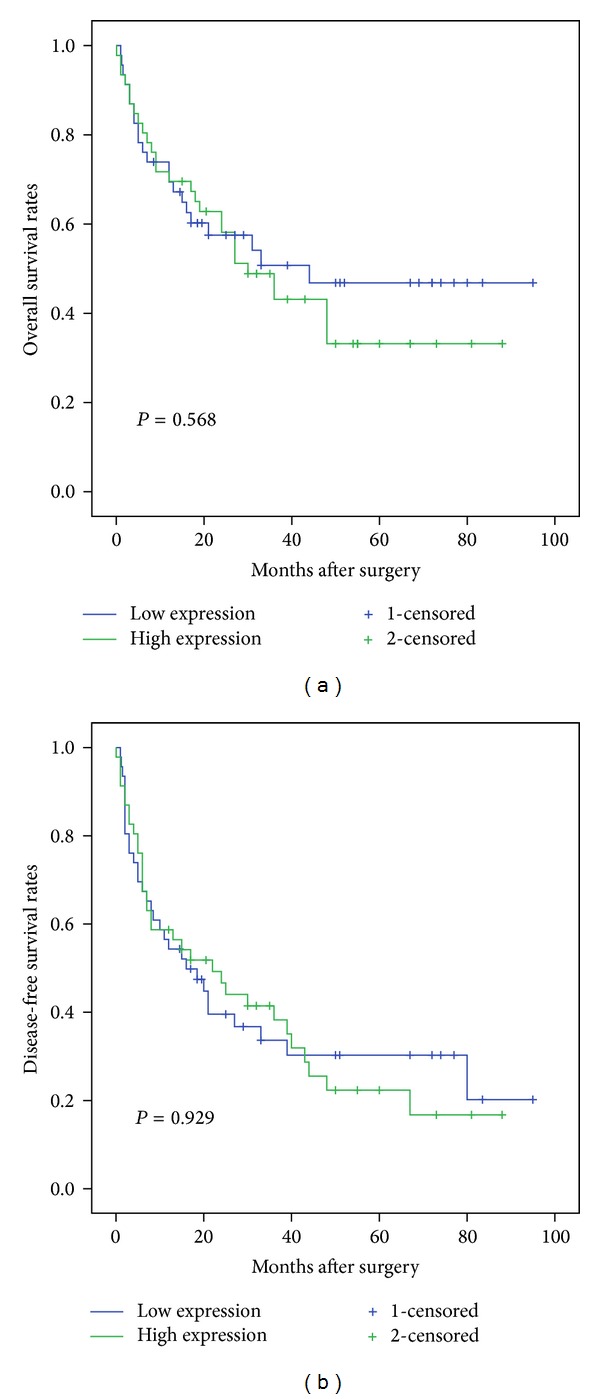
The correlation between miR-183 expression and the prognosis of HCC patients was analyzed by the Kaplan-Meier survival curve. The *P* value was calculated using the log-rank test between HCC patients with high and low miR-183 expression groups. (a) There was no significant difference in the overall survival rates of high-miR-183 expression group and low-miR-183 expression group (*P* = 0.568). (b) There was no significant difference between disease-free overall survival rates of high-miR-183 expression group and low-miR-183 expression group (*P* = 0.929).

Patients with HCC were divided into two groups by the median value of the level of miR-183. The Kaplan-Meier survival curves revealed that there were no significant differences in the overall survival and disease-free survival rates between high-miR-183 group and low-miR-183 group (log-rank test, *P* = 0.568, *P* = 0.929) (Figure 3).

The Cox regression analysis revealed that growth pattern of tumor (*P* = 0.007) and vein invasion (*P* = 0.008) were independent prognostic factors for HCC, but not for miR-183 (Table 2).

## 4. Discussion

miRNAs are deregulated in many kinds of cancers, and it has been found that the deregulation of miRNAs acts as oncogenes or tumor suppressors in cancer onset and progression [3]. In HCC, previous reports showed that the deregulated miRNAs may play different roles in HCC development and progression by kinds of mechanisms [13–20]. More importantly, the expressions of miRNAs were correlated with clinicopathological factors and prognosis of HCC patients. For example, let-7c was associated with the poor tissue differentiation in HCC patients [21]; miR-221 [22] and miR-22 [13] were correlated with prognosis of HCC.

Our preliminary study found that miR-183 expression was upregulated in HCC tumor tissue by RT2 miRNA PCR array [12]. In this study, qRT-PCR was used to analyse the expression of miR-183, and the result showed that miR-183 expression was significantly higher in benign liver diseases and HCC. Moreover, the results were consistent with those of Li et al. [9], Liu et al. [10], and Goeppert et al. [11]. The causes of upregulated miR-183 were reported that upregulation of miRNA can be due to amplification, deregulation of a transcription factor, or demethylation of CpG islands in the promoter regions of the gene [23]. In addition, miR-183 expression was significantly higher in HCC than in NC, CH, LC, and NT, which indicated that the regulatory mechanism of miR-183 is related to the progression of cancer onset.

The deregulated miR-183 may behave as oncogene in HCC. It was reported that miR-183 inhibited apoptosis in HCC cells by repressing the PDCD4 expression [9] or reduced the expression of tumor suppressor gene AKAP12 in human hepatocarcinogenesis [11]. In order to further discuss the function of miR-183 in the onset and development of HCC, we combined with the expression of miR-183 and clinicopathological factors of HCC patients. The result showed that the expression of miR-183 was higher in TNM stage (III-IV) than in TNM stage (I-II), prompting that miR-183 may promote the development of HCC. Furthermore, Budhu et al. [24] reported that the high level of miR-183 was associated with the poor prognosis of HCC patients; however, our results showed that miR-183 may not be an independent prognosis factor for HCC. Further research may help to clarify the role of miR-183 in prognosis of HCC.

Besides, the differentiating power in HCC patients and controls was also detected. And the results showed that miR-183 yielded an AUC of 0.808 with 59.8% sensitivity and 91.8% specificity. Furthermore, Liu et al. [10] reported that the levels of serum miR-183 expression were significantly lower in the postoperative samples than in the preoperative samples. Therefore, serum miR-183 may be used in the diagnosis of HCC, and combined together with other tumor markers, such as AFP, it might improve the sensitivity and specificity.

In summary, miR-183 expression was significantly higher in HCC patients, indicating that the regulatory mechanism of miR-183 is related to HCC. But, whether miR-183 could be a prognostic marker for HCC patients or not needs a further study. We will carry out a multicenter and large-scale study to confirm our results in the future.

## Figures and Tables

**Table 1:** Correlation between miR-183 expression and clinicopathological factors of HCC.

Factors	*n*	miR-183 level 2^−ΔΔCT^ *M* (*P*25~*P*75)	*r*	P*
Gender				
Male	79	9.178 (2.564~29.231)	−0.023	0.827
Female	13	6.431 (3.886~16.491)		
Age (years)				
≤55	50	8.415 (2.715~31.069)	−0.002	0.981
>55	42	10.543 (2.583~23.855)		
HBV				
+	72	8.880 (2.687~21.728)	−0.078	0.887
−	20	10.2773 (2.646~47.116)		
HCV				
+	6	12.556 (0.830~99.732)	−0.002	0.985
−	86	9.015 (2.715~23.855)		
Liver cirrhosis				
Yes	86	9.845 (2.761~29.437)	0.235	**0.025**
No	6	3.007 (1.606~4.862)		
Child-Pugh score				
A	77	8.583 (2.615~29.643)	0.046	0.661
B	15	10.920 (4.737~19.942)		
AFP (ng/mL)				
≤400	52	10.525 (2.588~26.252)	−0.040	0.705
>400	40	8.026 (2.799~28.121)		
Tumor number				
1	59	7.803 (2.961~33.794)	0.064	0.544
≥2	33	10.235 (3.989~24.257)		
Tumor size (cm)				
≤3	12	8.717 (2.799~29.403)	0.027	0.799
>3	80	10.547 (2.223~22.574)		
Vein invasion				
Yes	25	9.178 (4.228~19.525)	0.014	0.892
No	67	8.583 (1.564~33.794)		
TNM grade				
I-II	46	5.480 (2.381~11.794)	0.213	**0.042**
III-IV	46	11.795 (4.231~33.874)		
Tumor grade				
W	22	4.754 (1.656~68.768)	−0.060	0.365
M	48	10.047 (3.010~28.786)		
P	22	7.229 (2.190~11.677)		

*The Spearman correlation coefficients were used to analyze the correlation between miR-183 expression and clinicopathological factors; *P* < 0.05 was considered statistically significant; W: well differentiated; M: moderately differentiated; P: poorly differentiated; HBV: hepatitis B virus; HCV: hepatitis C virus; *r*: correlation coefficient; TNM: tumor node metastasis; AFP: alpha-fetoprotein.

**Table 2:** Univariate and multivariate analyses of prognosis factors associated with overall and disease-free survival rates in patients with HCC.

Factors	Overall survival			Disease-free survival		
	Univariate P*	Multivariate		Univariate *P*	Multivariate	
		HR (95% CI)	*P *		HR (95% CI)	P*
Gender (male/female)	NS	—	NA	NS	—	NA
Age (≤55/>55)	NS	—	NA	NS	—	NA
HCV (+/−)	NS	—	NA	NS	—	NA
HBV (+/−)	NS	—	NA	NS	—	NA
Liver cirrhosis (yes/no)	NS	—	NA	NS	—	NA
Child-Pugh score (A/B)	NS	—	NA	NS	—	NA
Tumor size (≤3/>3 cm)	NS	—	NA	NS	—	NA
Tumor number (1/≥2)	NS	—	NA	**0.001**	**1.211 (1.182–3.128)**	**0.006**
Growth pattern (expansive/invasive)	**0.000**	**3.006 (1.360–6.646)**	**0.007**	**0.001**	—	NS
Tumor grade (W/M/P)	NS	—	NA	NS	—	NA
TNM grade (I-II/III-IV)	**0.011**	—	NS	**0.006**		NS
AFP level (≤20/>20 ng/mL)	NS	—	NA	NS	—	NA
Vein invasion (yes/no)	**0.000**	**1.860 (0.929–3.724)**	**0.008**	**0.001**	**2.043 (1.061–3.934)**	**0.033**
miR-183 (high/low group)	NS	—	NA	NS	—	NA

*P < 0.05 was considered statistically significant; W: differentiated; M: moderately differentiated; P: poor differentiated; HBV: hepatitis B virus; HCV: hepatitis C virus; *r*: correlation coefficient; TNM: Tumor Node Metastasis; AFP: alpha-fetoprotein; HR: Hazard ratio; 95% CI: 95% confidence interval.
